# Supplementary material for: Comparative genomics provides new insights into the diversity, physiology, and sexuality of the only industrially exploited tremellomycete: Phaffia rhodozyma
Source: BMC Genomics. 2016 Nov 9;17:901. doi: 10.1186/s12864-016-3244-7 (PMC5103461; doi:10.1186/s12864-016-3244-7)
Supplement: Additional file 6: — List of orphan genes with links to PFAM (related to Additional file 1: Table S1). (ZIP 1428 kb) [file 12864_2016_3244_MOESM6_ESM.zip › PFAM_UNIQUE/G03616_P.html]

#                                                                            --- full sequence --- -------------- this domain -------------   hmm coord   ali coord   env coord
  
# target name        accession   tlen query name           accession   qlen   E-value  score  bias   #  of  c-Evalue  i-Evalue  score  bias  from    to  from    to  from    to  acc description of target
  
#------------------- ---------- ----- -------------------- ---------- ----- --------- ------ ----- --- --- --------- --------- ------ ----- ----- ----- ----- ----- ----- ----- ---- ---------------------
  
G03616\_P             -            356 DUF4643              PF15485.1    284       3.7    5.6   6.2   1   2       0.2   1.2e+03   -2.6   0.2    19    39    90   109    68   114 0.53 - Domain of unknown function (DUF4643)  
G03616\_P             -            356 DUF4643              PF15485.1    284       3.7    5.6   6.2   2   2   8.7e-05      0.52    8.4   1.6    93   166   230   302   178   324 0.87 - Domain of unknown function (DUF4643)
